# Phosphoenolpyruvate carboxykinases as emerging targets in cancer therapy

**DOI:** 10.3389/fcell.2023.1196226

**Published:** 2023-05-12

**Authors:** Yong Yu, Jingying Li, Kaiming Ren

**Affiliations:** ^1^ Department of Ophthalmology, Shengjing Hospital of China Medical University, Shenyang, Liaoning, China; ^2^ Department of Health Management, Shengjing Hospital of China Medical University, Shenyang, Liaoning, China; ^3^ Department of Thoracic Surgery, Shengjing Hospital of China Medical University, Shenyang, Liaoning, China

**Keywords:** metabolic reprogramming, phosphoenolpyruvate carboxykinase, gluconeogenesis, immunity, cancer

## Abstract

Metabolic reprogramming is commonly accompanied by alterations in the expression of metabolic enzymes. These metabolic enzymes not only catalyze the intracellular metabolic reaction, but also participate in a series of molecular events to regulate tumor initiation and development. Thus, these enzymes may act as promising therapeutic targets for tumor management. Phosphoenolpyruvate carboxykinases (PCKs) are the key enzymes involved in gluconeogenesis, which mediates the conversion of oxaloacetate into phosphoenolpyruvate. Two isoforms of PCK, namely cytosolic PCK1 and mitochondrial PCK2, has been found. PCK not only participates in the metabolic adaptation, but also regulates immune response and signaling pathways for tumor progression. In this review, we discussed the regulatory mechanisms of PCKs expression including transcription and post-translational modification. We also summarized the function of PCKs in tumor progression in different cellular contexts and explores its role in developing promising therapeutic opportunities.

## Introduction

Tumor metabolic remodeling is often accompanied by alterations in the expression of metabolic enzymes. In addition to catalyzing intracellular metabolic responses, metabolic enzymes are also involved in gene expression, cell cycle, DNA damage repair, antioxidant capacity, survival, proliferation, apoptosis and the regulation of tumor microenvironment, expanding the metabolic dependence of cancer cells and endows cancer cells with the capability to adapt to different environmental stimuli (Kim et al., 2019; Faubert et al., 2020; Cassim et al., 2020; Pan et al., 2021; DeBerardinis et al., 2022). Herein, further exploration of the role of these metabolic enzymes in tumor initiation and development, and the prospect of targeting these metabolic enzymes as new therapeutic strategies, which may provide new metabolic markers and molecular targets for tumor individualized therapy and great guiding significance for the development of targeted drugs in tumor metabolism (Cassim et al., 2019; Zhu et al., 2019; Pavlova et al., 2022).

**FIGURE 1:**
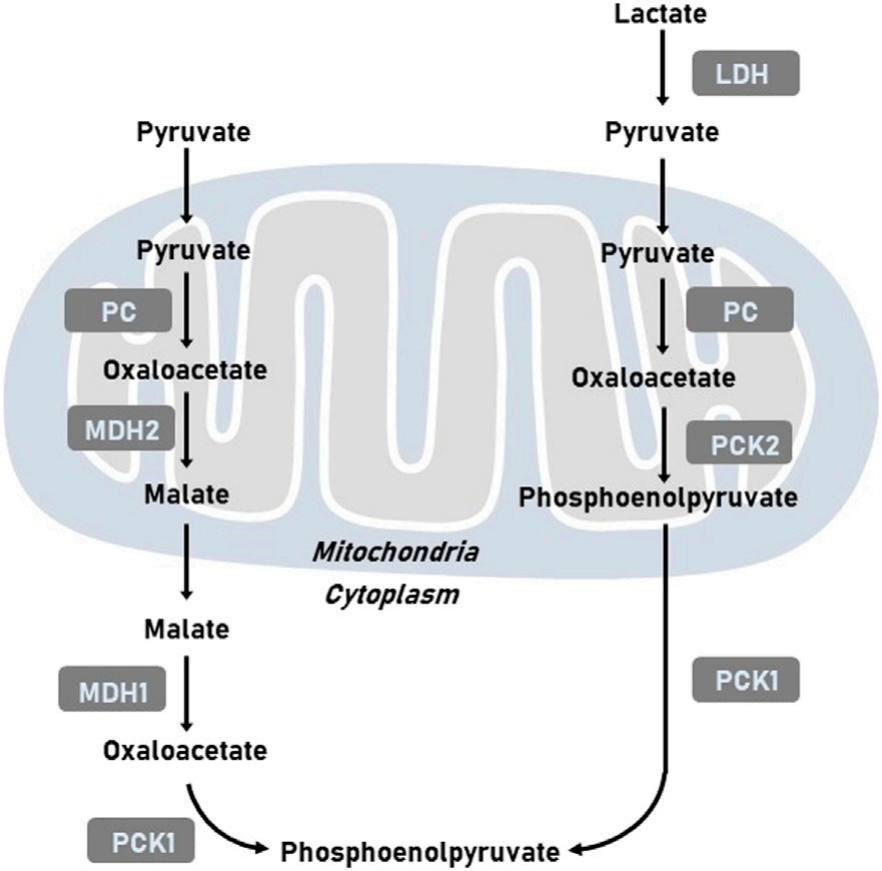
Representative images of metabolic pathways related to PCK. LDH, lactate dehydrogenase; MDH1, cytoplasmic malate dehydrogenase; MDH2, mitochondrial malate dehydrogenase; PC, pyruvate carboxylase; PCK1, cytoplasmic phosphoenolpyruvate carboxykinase; PCK2, mitochondrial phosphoenolpyruvate carboxykinase.

It has been well-established that tumor cells display elevated level of glycolysis in the presence of oxygen to support survival and proliferation of cancer cells. Gluconeogenesis, the reverse pathway of glycolysis, could antagonize tumor glycolysis depending on main enzymes, such as phosphoenolpyruvate carboxykinase (PCK), fructose-1,6-bisphosphatase (FBPase), and glucose-6-phosphatase (G6Pase). PCK mediates the conversion of oxaloacetate (OAA) and GTP into phosphoenolpyruvate (PEP), GDP, and CO_2_ (Yu et al., 2021). As the mitochondrial membrane is impermeable to OAA, OAA is initially converted to malate and then shuttled to the cytoplasm, where malate is re-oxidized into OAA. OAA is then incorporated into the gluconeogenesis via the production of PEP induced by PCK (Figure 1). The reaction catalyzed by PCK in gluconeogenesis incoporates the TCA cycle with glycolysis. Two isoforms of PCK have been reported, namely cytosolic PCK1 and mitochondrial PCK2. In addition to participate in metabolic adaptation, PCK regulates immune response and signaling pathways to mediates tumor initiation and development (Ho et al., 2015). In this review, we discussed the regulatory mechanisms of PCK expression including transcription and post-translational modification. We also summarized the function of PCK in tumor progression and explores its role in developing promising therapeutic opportunities.

## Regulation of PCK expression

The transcriptional level of PCK1 has been reported to be regulated by multiple factors, including the nuclear factor 1, cAMP response element, glucocorticoid response region, thyroid hormone response element, PPARγ-binding element, insulin/SREBP-1 response element (Yu et al., 2021). The regulatory mechanisms of PCK2 transcription are relatively limited. Purine element binding protein α, which participates in the regulation of oxidative phosphorylation and fatty acid metabolism, possess binding peaks in the promoter of PCK2 (Sun et al., 2020). Besides, PGC-1β and ERRα could enhance glutamine metabolism to support survival of colorectal tumor cells via transcriptional upregulation of PCK2 (Frodyma et al., 2022).

The post-translational modifications of PCK1 activity have also been reported. PCK1 activity is glucose-dependent and regulated by p300 and SIRT1. p300 mediates PCK1 acetylation for anaplerosis upon high glucose, whereas SIRT1 deacetylates PCK1 to replenishe gluconeogenesis under low glucose (Latorre-Muro et al., 2018). In addition, metabolic adaptation can also be regulated by acetylation of the Lys-491 residue of PCK2 and Lys-473 residue of PCK1 (PCK1-K473) by the lysine acetyltransferase 8 (KAT8), leading to isoenzyme transition from cytoplasmic PCK1 to mitochondrial PCK2 (Jing et al., 2021).

## PCK and immune response

Immune cells undergo different metabolic adaptation to satisfy its demands for energy and biosynthesis (Xia et al., 2021). It has been widely accepted that cellular metabolism is determinant for the function and homeostasis of immune cells. CD8^+^ memory T (Tm) cells are critical for anti-tumor immunity. PCK1-mediated glycogen metabolism is important for the formation and maintenance of Tm cells by altering reactive oxygen species (ROS) levels. Tm cells could increase PCK1 expression to increase glycogenesis via gluconeogenesis. Blocking PCK1 expression could increase ROS levels, impairing CD8^+^ Tm cell formation and maintenance (Ma et al., 2018). Acetyl-CoA is directed to ketogenesis in the mitochondria to regulate PCK1 expression. In CD8^+^ Tm cells, β-hydroxybutyrate produced from ketogenesis could epigenetically modify Lys 9 of histone H3 of Foxo1 and Ppargc1a via β-hydroxybutyrylation to upregulate the level of FoxO1 and PGC-1α, which could ultimately increase PCK1 expression to divert the carbon flow from gluconeogenesis to glycogen and the pentose phosphate pathway. These results reveal that PCK1 acts as an important metabolic link in CD8^+^ Tm cells, interwinding epigenetic modification and immune cell function (Zhang et al., 2020).

**FIGURE 2:**
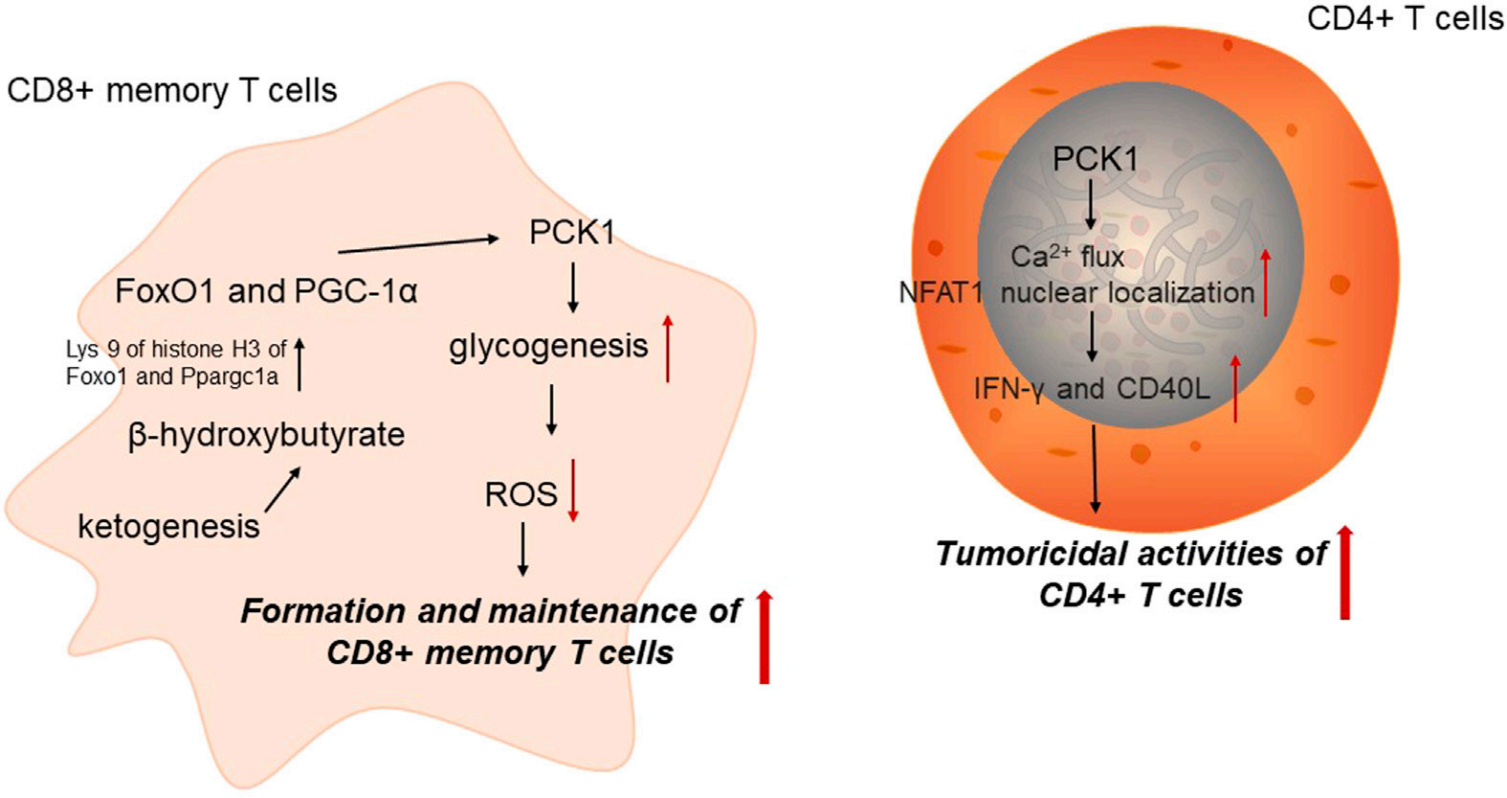
PCK1 and anti-tumor immunity. PCK1, cytoplasmic phosphoenolpyruvate carboxykinase; ROS, reactive oxygen species.

PEP has been identified as an important metabolite determining T cell function. As the key enzyme catalyzing the conversion of OAA into PEP, PCK1 overexpression could elevate PEP levels in T cells under glucose-limited conditions to enhance the anti-tumor effect of tumor-specific CD4^+^ T cells. Mechanically, PCK1 could reverse the effects of glucose limitation on Ca^2+^ flux and NFAT1 nuclear localization in CD4^+^ T cells, which reduced the reliance of both CD4^+^ and CD8^+^ T cells on glucose for Ca^2+^-NFAT1 pathways in glucose-poor conditions to increase production of IFN-γ and CD40L of Trp-1 CD4^+^ T cell. Therefore, PCK1 may be a promising therapeutic target involved in metabolic adaptation to increase effector functions of tumor-specific T cells in glucose-deprived environments (Ho et al., 2015) (Figure 2).

## PCK and multiple malignancies

### PCK and hepatocellular carcinoma

Hepatocellular carcinoma (HCC) is a dominating malignancy of the liver and one of the primary causes of cancer-related death worldwide. HCC cells reprogram their metabolism to satisfy their metabolic demand and survive under hypoxic and nutrient-deprived environment (Cassim et al., 2018). The expression of PCK1 has been detected to be significantly downregulated in HCC, and low PCK1 expression is correlated with shorter survival in HCC patients. PCK1 has been implicated in the TCA cycle through cataplerotic function. In glucose-limited HCC cells, PCK1 upregulation could drive TCA cataplerosis, contributing to energy crisis and oxidative stress. Decreasing ROS level or increasing TCA intermediate α-ketoglutarate could reduce cell death resulted from PCK expression (Liu et al., 2018).

PCK1 deficiency function as a link between metabolic reaction and post-translational modification. PCK1 deficiency promotes hexosamine biosynthetic pathway (HBP)-induced protein O-GlcNAcylation in HCC cells, which enhances tumor proliferation. Mechanistically, PCK1 inhibition markedly induces its O-GlcNAcylation to impairs KAT5 ubiquitination and maintain KAT5 stabilization. KAT5 O-GlcNAcylation could epigenetically activate TWIST1 and increase MMP9 and MMP14 expression by c-Myc acetylation, ultimately enhancing epithelial-mesenchymal transition (EMT) in HCC (Liu et al., 2021). PCK1 silencing also enhances CHK2 O-GlcNAcylation and HCC growth under low glucose conditions. PCK1 inhibition increases the global O-GlcNAcylation levels upon glucose deprivation. PCK1 deficiency in HCC cells leads to elevated OAA level and increased *de novo* uridine triphosphate synthesis contributing to uridine diphosphate-N-acetylglucosamine (UDP-GlcNAc) biosynthesis (Xiang et al., 2021).

In addition to its role in epigenetic regulation, PCK1 silencing also contributes to HCC initiation and development by regulating downstream signaling pathways. Hepatitis B X-interacting protein (HBXIP) function as an oncoprotein by modulating PCK1-mediated gluconeogenesis and glucose metabolism reprogramming. HBXIP attenuated PCK1 expression through inhibiting the activation of FOXO1 in HCC cells, and increased miR-135a targeting the 3′UTR of FOXO1 mRNA. PCK1 overexpression could reverse the HBXIP-increased growth of HCC cells. Thus, HBXIP could impair the gluconeogenesis through inhibiting PCK1 activity to enhance HCC development (Shi et al., 2016). PCK1 deficiency could also promote HCC cell proliferation by inactivating AMPK, inhibiting p27Kip1 expression, and induce CDK/Rb/E2F activation, which supports cell cycle transition from the G1 to S phase upon low glucose stimulation (Tuo et al., 2019).

Notably, the protein kinase activity of PCK1 has been recognized in HCC cells. It has been demonstrated that activated AKT mediates the phosphorylation of cytosolic PCK1 at Ser90 in HCC cells. Phosphorylated PCK1 further translocated to the endoplasmic reticulum (ER), leading to INSIG1 phosphorylated at Ser207 and INSIG2 at Ser151. Current understanding indicated that tumor cells rely on lipogenesis to support tumor proliferation, and sterol regulatory element-binding proteins (SREBPs) are required for mediating lipogenesis. It is worth noting that SREBPs are attenuated by a complex comprised by INSIGs, sterols and SREBP cleavage-activating protein (SCAP) in the ER. This phosphorylation of INSIGs by phosphorylated PCK1 could reduce the binding of sterols to INSIGs and impairs the interplay between INSIGs and SCAP, contributing to the activation of SREBPs and downstream lipogenesis-related genes, and tumor proliferation (Xu et al., 2020).

Sorafenib has been approved to be the first-line targeted regimens for advanced liver cancer, and acquired resistance is the primary obstacles for these patients. Intriguingly, sorafenib leads to acetylation alterations towards a different metabolic phenotype. Acetylation could induce isoenzyme transition from PCK1 to PCK2, therefore resulting in tumor resistance to sorafenib. Specifically, acetylation of the Lys-491 (K491) residue of PCK2 and Lys-473 (K473) residue of PCK1 mediated by KAT8 contributes to isoenzyme transition from PCK1 to PCK2. KAT8-mediated PCK2 acetylation at K491 impairs lysosomal degradation to elevate PCK2 expression in sorafenib-resistant liver tumor cells, while PCK2 silencing in sorafenib-resistant cells could reduce tumor resistance. In particular, upregulated PCK2 expression was associated with poor prognosis in patients who received sorafenib. Thus, blocking acetylation-mediated isoenzyme transition from PCK1 to PCK2 may be a promising anti-tumor strategy to conquer tumor resistance to sorafenib in liver cancer (Jing et al., 2021).

### PCK and lung cancer

Lung cancer is the primary cause of cancer-related mortality (Sung et al., 2021). Intriguingly, increased PCK2 expression has been detected in tumor tissue of non-small-cell lung cancer patients (Vincent et al., 2015). It has been well-established that tumor microenvironment (TME) has been implicated in the initiation and development of lung cancer. In the TME, the complex interplays between diverse cells could shape the metabolic features of the TME, indicated by hypoxic, acidic and glucose-limited conditions. PCK2 expression and catalytic activity are increased by low-glucose conditions. Within the glucose-deprived TME, PCK2 exerts crucial effects to fuel tumor growth. Depending on the catalytic activity of PCK2, glucose deprivation led to enhanced conversion from glutamine to PEP. Further, PEP support the biosynthesis of serine and purine (Vincent et al., 2015). PCK2 also functions as a key regulator of oxidative phosphorylation (OXPHOS) and redox balance in nutrient-limited lung tumor cells. Under nutrient-deprived condition, PCK2 inhibition elevated TCA cycle intermediates, OXPHOS and glutathione oxidation. Increased carbon flow to TCA cycle by PCK2 silencing impaired growth of lung tumor under starvation (Bluemel et al., 2021). Under nutrient-deprived conditions, 13C-labeled analysis identified lactate as a gluconeogenic precursor to the production of PEP catalyzed by PCK1 in lung tumor cells. Small interfering RNA targeting PCK2 or the pharmacological inhibitor 3-mercaptopicolinate could increase low glucose-induced cell apoptosis in lung tumor cells (Leithner et al., 2015).

### PCK and melanoma

Melanoma is a skin cancer caused by a malignancy of melanocytes (Ahmed et al., 2020). Stem cells that could repopulate tumors, named tumor-repopulating cells (TRCs) (Jiang et al., 2020). TRCs are a tumorigenic sub-population of tumor cells that drives tumorigenesis. TRCs derived from melanoma could alter glucose metabolism by hijacking PCK1. Increased PCK1 expression in TRCs enhances serine and glycerol-3-phosphate metabolism rather than mediating gluconeogenesis. PCK1 inhibition could block the growth of TRCs and impair the development of melanoma. Collectively, targeting PCK1 upregulation in TRCs derived from melanoma may be a promising anti-tumor strategy (Li et al., 2015). In contrast to the tumor-promoting effects of PCK1 in melanoma, PCK2 downregulation has been indicated as a metabolic characteristic of TRCs from melanoma. PCK2 participates in the regulation of glucose carbon flow direction in TRCs. Silencing PCK2 increases incorporation of citrate from mitochondria to the cytosol, contributing to decreased carbon flow of TCA cycle and OXPHOS in TRCs (Luo et al., 2017). PCK2 overexpression could impair TRC growth and block tumor progression of melanoma. Notably, PCK2 downregulation acts as a metabolic feature of TRCs from melanoma, which may bring novel therapeutic targets for the treatment of melanoma.

### PCK and breast cancer

Breast cancer has now surpassed lung cancer to become the most common malignant tumor worldwide (Sung et al., 2021). There are 2.26 million newly diagnosed cases of breast cancer each year, accounting for 11.7% of all tumors. Hypoxia is a common feature of breast tumor microenvironments. In breast tumors, hypoxia could mobilize transcription factors HIF1α and FoxO1 to mediate epigenetically upregulate PCK1. PCK1 further induced retrograde carbon flow away from gluconeogenesis, leading to decreased glutathione level and elevated ROS production to support hypoxic breast TRC growth (Tang et al., 2021). Breast cancer is categorized into diverse subtypes based on gene expression profiles. Basal-like breast cancer, an aggressive subtype of breast cancer, exhibit poor prognosis and show resistance to chemotherapy, which may result from the existence of breast cancer stem cells. Intriguingly, PCK2 participates in the regulation of basal-like breast tumor stemness and OXPHOS. Recent studies demonstrated that reduced PCK2 expression blocked OXPHOS and elevated tumor stemness exerted by miR-200c in p53 mutation-bearing basal-like breast tumor cells. Thus, silencing of the p53-miR-200c-PCK2 axis could bring metabolic adaptaion for tumor stemness, resulting in the development of basal-like breast tumors (Chao et al., 2021).

### PCK and prostate cancer

Castration-resistant prostate cancer (CRPC) are commonly accompanied by neuroendocrine differentiation, which lacks effective therapeutic strategies (Wang et al., 2021). Intriguingly, targeting PCK1 has been found to attenuate the neuroendocrine phenotype of CRPC and impair the growth of these prostate tumors. Worth noting, ZBTB46-leukemia inhibitory factor (LIF) axis is critical for PCK1-induced glucose metabolism and neuroendocrine differentiation of CRPC, which may be exploited as therapeutic targets for blocking CRPC development. Once LIF signaling is activated, PCK1 expression is regulated by the ZBTB46 transcription factor. PCK1 could enhance prostate tumor cell proliferation and reciprocally elevates ZBTB46 levels to upregulate the expression of neuroendocrine markers. LIF/ZBTB46 axis is a key mechanism for PCK1-driven glucose metabolism, which may bring improvements in prostate cancer treatment after androgen deprivation therapy using PCK1 inhibitors (Liu et al., 2019; Wen et al., 2022). Additionally, elevated PCK1 levels are correlated with prostate tumor progression by normal prostate-derived stromal cells (Peng et al., 2011). These findings indicated PCK as a promising therapeutic target for the treatment of CRPC.

### PCK and colon cancer

Under nutrient-limited conditions, colon tumor cells tend to enhance the consumption of glutamine or other nutrients, enabling colon tumor cells to satisfy their energetic demand for survival and proliferation. In colon tumors, PCK1 has been found to enhance glucose and glutamine consumption toward anabolic metabolism to promote colon tumor cell proliferation. PCK1 also participates in the synthesis of ribose from glutamine (Montal et al., 2015). PCK1 silencing also led to increased energy crisis indicated by reduced ATP production and elevated AMPK activation. 13C tracing has revealed that the PCK1 inhibitor reduced the incorporation of lactate into the TCA cycle in colon tumor cells. Based on these results, PCK1 functions as a metabolic link to support colon cancer cell proliferation. PCK1 also contributes to liver metastatic colonization of colorectal tumor by increasing pyrimidine nucleotide biosynthesis under hypoxia (Yamaguchi et al., 2019). Pharmacologic inhibition of PCK1 could blocked the liver metastatic colonization of colorectal tumors (Yamaguchi et al., 2019).

## Conclusion

PCK, the rate-limiting enzymes in gluconeogenesis, play multifaceted roles in tumor initiation and development. It is worth noting the that the diversity of PCK expression in different cancer models make it a challenging therapeutic target, and it is required to fully investigate the underlying molecular mechanisms of PCK-mediated tumor development in different tissues and disease stages. To identify types of tumors that can be effectively treated with PCK-targeted therapy, it is imperative to develop predictive biomarkers for the response of tumor cells for PCK-targeted therapy. Intriguingly, PCK-targeted therapy may exert enhanced tumoricidal effect when combined with other traditional therapies. There are growing evidences demonstrated that PCK acts as a metabolic regulator for immune response of tumors, suggesting that combinational regimens of PCK-targeted therapy and immune checkpoint inhibitors may be promising strategies for the treatment of tumors.
